# Factors Influencing Administration, Recognition, and Compliance of Medicine among Community Residents from Jilin Province, China: A Questionnaire Study

**DOI:** 10.1155/2020/8730212

**Published:** 2020-07-18

**Authors:** Yan-Qing Song, Wen-Rui Zhang, Si-Xi Zhang, Ting-Hong Wang, Zhe Jiang, Chun-Feng Meng, Tian-Zhen Zhang, Qi-Zhong Xin, Ke-Jun Cao, Yue Zhang, Ying Sun

**Affiliations:** ^1^Department of Pharmacy, The First Hospital of Jilin University, Changchun, Jilin 130021, China; ^2^Chaoyang District Hospital of Changchun City, Changchun, Jilin 130021, China; ^3^Department of Pharmacy, Affiliated Hospital of Yanbian University, Yanji, Jilin 133000, China; ^4^Department of Pharmacy, People's Hospital of Changchun City, Lvyuan District, Changchun, Jilin 130021, China; ^5^Department of Pharmacy, The Central Hospital of Meihekou City, Changchun, Jilin 130600, China; ^6^Department of Pharmacy, Shuangyang District Hospital of Changchun City, Changchun, Jilin 130600, China; ^7^Department of Pharmacy, People's Hospital of Jiutai District, Changchun, Jilin 130000, China; ^8^Drug Clinical Trial Institution, The First Hospital of Jilin University, Changchun, Jilin 130021, China; ^9^Changchun Medical College, Changchun, Jilin 130031, China

## Abstract

**Introduction:**

To identify and analyze factors that influence administration, recognition, and compliance of medicine among community residents in Jilin Province, China.

**Methods:**

A survey was carried out among 2417 community residents in Jilin Province, China, to study their administration (CRA), recognition (CRR), and compliance (CRC) of medicine. Multivariate logistic regression analyses and chi-squared tests were performed to assess factors influencing CRA, CRR, and CRC.

**Results:**

Logistic analyses showed that gender, educational level, and occupation were influencing factors on CRA; age, educational level, smoking status, and health condition were influencing factors on CRR; and gender, age, occupation, and health condition were influencing factors on CRC.

**Conclusions:**

CRA, CRR, and CRC are associated with specific lifestyles and social economic statuses of community residents. Attention should be paid to influencing factors in order to facilitate community pharmaceutical care, promote the rational use of drugs, and ensure the safe use of medications. This study explores the type and extent of professional services provided through community pharmacies in Jilin Province, China, and provides evidence for optimizing the quality of community pharmacy services.

## 1. Introduction

The World Health Organization (WHO) defines adverse drug reactions (ADRs) as “any response to a drug that is noxious and unintended and occurs at doses normally used in man for prophylaxis, diagnosis, or therapy of diseases” [1]. ADRs are a major global clinical problem and can lead to substantial mortality and morbidity [2]. ADRs are caused by a combination of patient-related, medication-related, and social-related factors [3]. Potentially inappropriate medications (PIMs) refer to the use of a medication whose adverse outcome outweighs the potential benefit [4].

Risk factors of medication-related represented patient-, environment-, and treatment-related domains, which can be identified at home visits, include poor adherence with medication regimens, inappropriate storage of medication, use of expired medication, application/usage of inappropriate medications, and possible underdosing or possible overdosing. As medication use is an effective way of providing basic medical and health services, rational administration of pharmacy services has become a critical factor influencing the quality of community health services.

Pharmacies are an important part of health care in China. In the 1990s, pharmaceutical care was first introduced in China, and the responsibility of the pharmacist was defined as improving the patient's quality of life by providing health care services and achieving specific results. According to the European Society of Clinical Pharmacy, one of a clinical pharmacist's responsibilities is the detection and prevention of harmful medication errors [5–9] and drug-related problems, including ADRs [10–12]. Before taking a prescription drug, patients are counseled by their physician or clinical pharmacist with drug-related questions. For community residents, the use of medications is becoming more complex as more medicines are being introduced to the market, so the demand for community-based pharmaceutical care is increasing.

However, current knowledge on the scope and quality of pharmacy services in developing countries is limited. Therefore, it is worthwhile to further investigate the current status of community residents' administration (CRA), recognition (CRR), and compliance (CRC) of medicine in China. We studied this topic in randomly selected communities in Jilin Province of China with the aim of providing a more effective service for residents and improving the public's knowledge of self-medication safety.

## 2. Methods

### 2.1. Population

A questionnaire survey was conducted between June 2016 and January 2017 in Jilin Province, China. A total of 2800 community residents (aged 18 years or older) completed the questionnaires. “Community residents” were defined as people living in homes or apartments of a community and not in institutions such as hospitals. Inclusion criteria included community residents having a clear understanding of the study, having no problems in communicating with investigators, being willing to cooperate with the researcher, and filling at least three or more prescriptions at a pharmacy in the past 12 months. Exclusion criteria were the submission of incomplete questionnaires and/or duplicated answers to each question on each page.

Authors summarized the problems pertaining to the rational use of drugs regarding CRA, CRR, and CRC when designing the questionnaire. The survey was anonymous. According to the results of the investigation and feedback, further argumentation, modifications, and finalization were undertaken by pharmaceutical experts. The survey providers randomly distributed the questionnaire to community residents who were asked to fill in the required information and submit the questionnaires on the spot. The Ethics Committee of the First Hospital of Jilin University approved the study protocol on May 2016 (2016-028), and written informed consent was obtained from all community residents to allow the use and publication of their data in the study.

### 2.2. Questionnaire Setting

The questionnaire included the following information: sociodemographic characteristics of the residents (including gender, age, educational level, occupation, monthly income, marital status, hospitalizations per year, medical insurance, types of medicines per day, number of medications received each time, adverse reactions, smoking status, alcohol consumption status, and self-rated health status); CRA (category of drug storage, storage method, whether it is classified, whether the medicine cabinet was cleaned regularly, and whether the individual has the knowledge of drug administration); CRR (understanding basic drug information including drug name, labelled indications, dosage, drug-drug interactions, drug-food interactions, and adverse effects); and CRC (drug intake behavior and strict adherence to the prescribed medicine and required dosage). The data were double entry and were independently checked by two investigators.

An analysis of questionnaire reliability was undertaken using Cronbach's alpha, which gave results for CRA, CRR, and CRC of 0.834, 0.782, and 0.825, respectively. Questionnaire scores were grouped in the following categories. CRA: poor (≤24 points) and good (25–31 points); and CRR: poor (≤28 points) and good (29–38 points). CRC scores could range from 0 to 8 with low adherence defined as a score < 6.

### 2.3. Statistical Analyses

SPSS v17.0 (SPSS Inc., Chicago, IL, USA) software package was used for statistical analyses. To examine bivariate associations between independent variables with poor and better CRA, CRR, and CRC, we performed chi-squared (*χ*^2^) analyses or Fisher's exact tests for categorical independent variables (*p* < 0.05 for statistical significance) and Mann-Whitney *U* test or *t*-test for continuous independent variables (*p* < 0.05 for statistical significance). Significant independent variables from bivariate analyses were further examined in multivariate logistic regression analysis to determine factors associated with poor and better categories of CRA, CRR, and CRC. A two-sided *p* value less than 0.05 was considered statistically significant.

## 3. Results

### 3.1. Sociodemographic Characteristics

**Figure 1:**
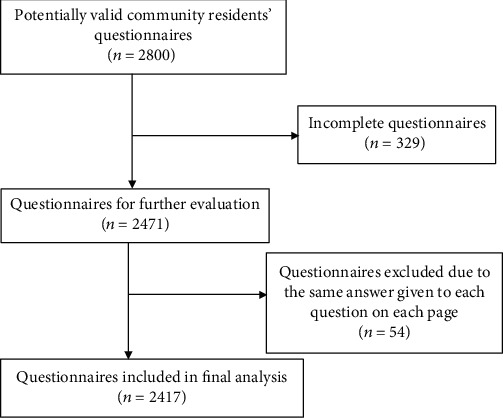
Flowchart showing the selection of valid questionnaires.

A total of 2800 questionnaires were distributed to the community residents, and 2417 usable questionnaires were returned (from 972 males and 1445 females) thus giving a valid response rate of 86.3%.  Figure 1 shows the details of the selection process. The average age of the community residents was 48.65 ± 15.06 years; 31.6% graduated from senior high school or polytechnic and 84.6% had a monthly income of less than $600, 73.7% were never smokers, and 66.6% were abstainers (Table 1). Community residents had some general knowledge of medication safety. Only about 10% of the residents had private medical insurance or other means to buy medications.

### 3.2. Influencing Factors on CRA, CRR, and CRC

The appropriateness of CRA, CRR, and CRC was 53.6%, 47.7%, and 54.0%, respectively; only 25.2% of the residents met the three criteria of CRA, CRR, and CRC. In the univariate analyses (Tables 2 and 3), we found that gender, age, educational level, and occupation of residents were significantly associated with appropriate CRA (*p* < 0.05). Gender, age, educational level, occupation, smoking status, and drinking levels were statistically significant (*p* < 0.05) when associated with appropriate CRR while gender, age, occupation, medical insurance, number of medications taken on each occasion, types of medicines per day, smoking status, health condition, and drinking levels of community residents were statistically significant (*p* < 0.05) when associated with appropriate CRC.

After controlling for potential covariates in the multivariable regression models (Table 4), we found that female gender, higher educational level (junior college and above), and professional and service provider occupations were significantly associated with a higher occurrence of appropriate CRA. Educational level (junior college, undergraduate, and above) and poor health condition (poor) were significantly associated with the raised occurrence of appropriate CRR. We also found that age and smoking status were negatively associated with the occurrence of appropriate CRR; these community residents did not generally understand the problems associated with drug use. Female gender, age, occupation, and health condition were positively associated with the occurrence of appropriate CRC, and these patients had better medication adherence.

## 4. Discussion

In this study, we measured the prevalence of potential medication-related risk factors among community residents. We found that the appropriateness of CRA, CRR, and CRC was 53.6%, 47.7%, and 54.0%, respectively. CRA, CRR, and CRC are associated with specific lifestyles and social economic statuses of community residents. Attention should be paid to factors that affect foster community pharmaceutical care, promote the rational use of drugs, and ensure the safety of medications.

According to the results of the multivariate logistic regression analysis, female community residents exhibited appropriate CRA and CRC. The compliance rate was significantly lower among males and those who were illiterate. Okumura et al.'s study showed that women were mainly responsible for the management of medications in the family household [13]. This underlines the important role of women in all aspects of health care, including as the main decision-maker for the management of stored medications. Indeed, females in China are generally obliged to be responsible for the health of their families. We also found that older age was associated with a lack of CRR. Access to CRR is not limited to doctors or pharmacists; some may come from the Internet or written instructions. Older individuals are more likely to suffer from visual or cognitive impairment and thus may find it more difficult to view product labels than their younger counterparts [14]. Age may limit the information acquisition. Regarding CRC, older age corresponded to increased compliance with drug therapy, although this is contrary to the results of another study [15] and may be due to geographical variation in participants' education as well as other social factors. Ageing is arguably associated with a higher awareness of health and drug relationships and stronger perceived drug effectiveness. Yet, the ability to read medication-related information via the Internet or written instructions can be reduced by factors such as visual impairment. Thus, age-related functional decline may curb access to medication-related information. This finding highlights the importance of providing easily accessible medication information to elderly people. Additionally, aged community residents with poorer health have a higher motivation to receive medications and had better compliance and paid more attention to their health problems since they had a stronger desire to improve their own health. However, community residents with very poor health status have less cognitive ability and are often over the age of 75 years, so age has an effect on CRR.

Moreover, a higher level of education also exerts a significant influence on CRA and CRR. A national study in China indicated that diabetes patients with higher education have a more positive attitude towards their diabetes and tend to achieve better blood glucose control [16]. Indeed, highly educated people are generally more capable of receiving and handling knowledge [16]. Conversely, according to the investigation, patients with a lower educational background were more likely to have the misconception that hypoglycemia was not a concern for diabetic patients, which hinders the adoption and implementation of ADR prevention and drug management.

Furthermore, community residents with better educational qualifications have a better knowledge of medication use. Indeed, education plays an important role in improving appropriate CRR knowledge. In line with education, we found that professional and service provider occupations were positively correlated with appropriate CRA and CRR, and such patients were better able to manage their drugs. This might be explained by their history of communicating with medical professionals during their working lives.

Recently, there has been a steady increase in the consumption of both over-the-counter (OTC) and prescription drugs. Drugs are often stored for long periods at the community resident's home, and leftover medication may later be used for self-medication. Moreover, the use of multiple drugs increases the risk of several drug-related problems. A recent study showed that only 7% of patients stored these drugs continuously at temperatures specified in the product label [17].

Proper storage temperatures are important because improper storage temperatures can lead to chemical instability resulting in a reduction in the effectiveness and a potential increase in adverse effects [18]. Patients are expected to store their drugs at home according to the storage conditions stated on the labels—such as in the case of drugs requiring refrigeration or storage in the original (outer) packaging to protect from moisture or light—which are provided by the drug companies in the package insert and on the drugs' packaging. In addition to adequate storage conditions, patients should use the drug before the expiry date and keep the drug in its undamaged primary package to ensure drug quality. Furthermore, adequate storage practices also require patients to have access to drug information, by having drugs stored that are identifiable (e.g., for caregivers) and having package inserts available [19–21].

Pharmacists deal with a broad variety of customers, some of whom lack awareness of potential drug interactions [22, 23] or potential side effects [24]. In addition, users of OTC drugs may confuse concepts such as “contraindications” with “side effects,” be unable to calculate simple dosages [25], or significantly overestimate the risk of side effects [26]. These errors result in PIMs or dosing of OTC drugs during hospitalizations, many of which are preventable [27]. Since OTC drugs have become commonly available, the primary way for community residents to obtain information about drugs is from the medication label, and patient compliance reportedly varies in the management of chronic diseases such as diabetes mellitus [15, 28]. Patient noncompliance with prescribed hypoglycemic medications may decrease treatment effectiveness [29, 30].

An attempt was made in this present study to measure the association of various sociodemographic factors and other patient characteristics against the compliance to medications and the reasons behind the nonadherence, as reported by the community residents. In our study, the compliance rate with drugs was found to be 54.0%. Two studies reported that compliance among diabetes patients (in all settings) in taking hypoglycemic drugs was 32.1% and 57.7%, respectively [15, 31]. However, among community residents, medication compliance rates were likely to be worse due to less advice being available from clinical pharmacists and doctors.

Some study limitations should be acknowledged. A potential source of bias is that those with less education and literacy may be less likely to participate in online surveys. However, for this hard copy questionnaire, the overall participation rate was high (*n* = 2800). Furthermore, the study focused on adherence in general, rather than specific conditions or medications. Further larger-scale studies using a longitudinal design are warranted to provide a more accurate demonstration of the factors influencing different diseases or different kinds of drugs. Finally, the study was restricted by the survey length, which limited the researchers' ability to explore specific disorders and identify each resident's health status in more detail. Although the results are representative only of Jilin Province in China, the study was highly consistent with other studies and thus may indirectly contribute to our general understanding of CRA, CRR, and CRC.

## 5. Conclusions

In conclusion, this study identified many inappropriate practices among community residents that could contribute to the improper use of medications. As community pharmaceutical care is a critical part of the community health service that can provide medicine in a safe, reasonable, and effective way, it should be customized to specific groups such as males, elderly people, and individuals with low education levels and less professional training. Large multicenter studies are required to explore the cost-effectiveness of community pharmacy services and the impact of these services on CRA, CRR, and CRC of medications.

## Figures and Tables

**Table 1:** Baseline characteristics of community residents.

Variables	Community residents (*n* = 2417)	Weighted percentages
*Gender*		
Male	972	40.2
Female	1445	59.8
*Age (years)*		
<18	1	0.1
18–40	797	33.0
41–60	1011	41.8
61–80	593	24.5
>80	15	0.6
*Ethnicity*		
Han Chinese	2206	91.3
Other	211	8.7
*Highest educational level*		
Primary school	177	7.3
Middle school	621	25.7
Senior high school or polytechnic	763	31.6
Junior college	537	22.2
Undergraduate	319	13.2
*Occupation*		
Agricultural worker	229	9.5
Industrial (or skilled) worker	817	33.8
Professional	385	15.9
Service provider	546	22.6
Other	440	18.2
*Monthly income*		
≤1000 RMB	182	7.5
1001–2000 RMB	661	27.3
2001–3000 RMB	672	27.8
3001–4000 RMB	530	21.9
4001–5000 RMB	241	10.0
>5000 RMB	131	5.4
*Marital status*		
Unmarried	280	11.6
Married	2063	85.4
Divorced	74	3.1
*Hospitalizations per year*		
0	1466	60.7
1	599	24.8
2–3	300	12.4
4–6	38	1.6
>6	14	0.6
*Medical insurance*		
Medical insurance in urban area	746	30.9
Urban employees' medical insurance	1025	42.4
“New-style” rural cooperative medical care	404	16.7
Commercial medical insurance	38	1.6
Private expenses	167	6.9
Others	37	1.5
*Types of medicines per day*		
≤2	1811	74.9
2–5	570	23.6
6–10	32	1.3
>10	4	0.2
*Number of medications received each time*		
≤5	1986	82.2
6–10	372	15.4
11–15	45	1.9
>15	14	0.6
*Adverse reactions*		
Yes	527	21.8
No	1417	58.6
Uncertain	473	19.6
*Smoking status*		
No smoking	1781	73.7
Light smoking (PI ≤ 200)	346	14.3
Middle-heavy smoking (PI 200–400)	199	8.2
Heavy smoking (PI ≥ 400)	30	1.2
Smoking cessation	38	1.6
Smoking cessation (≥2 years)	23	1.0
*Alcohol consumption status*		
No drinking	1609	66.6
Heavy drinking	52	2.2
Middle–heavy drinking	232	9.6
Light drinking	512	21.2
Temperance	9	0.4
Temperance (≥2 years)	3	0.1
*Self-rated health status*		
Very good	336	13.9
Good	944	39.1
Fair	924	38.2
Poor	198	8.2
Very poor	15	0.6

**Table 2:** Bivariate chi-squared analyses of categorical independent variables versus poor and better CRA, CRR, and CRC.

Variables	Community residents *N* (%)	CRA				CRR				CRC			
		Appropriately *N* (%)	Inappropriately *N* (%)	Statistics	*p*	Appropriately *N* (%)	Inappropriately *N* (%)	Statistics	*p*	Appropriately *N* (%)	Inappropriately *N* (%)	Statistics	*p*
*Gender*													
Male	972 (40.2)	520 (53.5)	452 (46.5)	*χ* ^2^: 25.816	<0.001	564 (58.0)	408 (42.0)	*χ* ^2^: 21.386	<0.001	466 (47.9)	506 (52.1)	*χ* ^2^: 2.450	0.117
Female	1445 (59.8)	621 (43.0)	824 (57.0)			700 (48.4)	745 (51.6)			646 (44.7)	799 (55.3)		
*Ethnicity*													
Han Chinese	2206 (91.3)	1165 (52.8)	1041 (47.2)	*χ* ^2^: 0.003	0.955	1060 (48.1)	1146 (51.9)	*χ* ^2^: 3.591	0.058	1193 (54.1)	1013 (45.9)	*χ* ^2^: 0.077	0.781
Others	211 (8.7)	111 (52.6)	100 (47.4)			87 (41.2)	124 (58.8)			112 (53.1)	99 (46.9)		
*Highest educational level*													
Primary school	177 (7.3)	105 (59.3)	72 (40.7)	*χ* ^2^: 80.087	<0.001	106 (59.9)	71 (40.1)	*χ* ^2^: 80.725	<0.001	90 (50.8)	87 (49.2)	*χ* ^2^: 7.226	0.124
Middle school	621 (25.7)	356 (57.3)	265 (42.7)			395 (63.6)	226 (36.4)			267 (43.0)	354 (57.0)		
Senior high school or polytechnic	763 (31.6)	358 (46.9)	405 (53.1)			412 (54.0)	351 (46.0)			349 (45.7)	414 (54.3)		
Junior college	537 (22.2)	227 (42.3)	310 (57.7)			223 (41.5)	314 (58.5)			266 (49.5)	271 (50.5)		
Undergraduate	319 (13.2)	95 (29.8)	224 (70.2)			128 (40.1)	191 (59.9)			140 (43.9)	179 (56.1)		
*Occupation*													
Agricultural worker	229 (9.5)	142 (62.0)	87 (38.0)	*χ* ^2^: 55.406	<0.001	141 (61.6)	88 (38.4)	*χ* ^2^: 38.197	<0.001	125 (54.6)	104 (45.4)	*χ* ^2^: 36.657	<0.001
Industrial (or skilled) worker	817 (33.8)	437 (53.5)	380 (46.5)			480 (58.8)	337 (41.2)			352 (43.1)	465 (56.9)		
Professional	385 (15.9)	152 (39.5)	233 (60.5)			176 (45.7)	209 (54.3)			136 (35.3)	249 (64.7)		
Service provider	546 (22.6)	221 (40.5)	325 (59.5)			254 (46.5)	292 (53.5)			284 (52.0)	262 (48.0)		
Other	440 (18.2)	189 (43.0)	251 (57.0)			213 (48.4)	227 (51.6)			215 (48.9)	225 (51.1)		
*Monthly income*													
≤1000 RMB	182 (7.5)	85 (46.7)	97 (53.3)	*χ* ^2^: 9.147	0.103	81 (44.5)	101 (55.5)	*χ* ^2^: 11.021	0.051	91 (50.0)	91 (50.0)	*χ* ^2^: 5.534	0.354
1001–2000 RMB	661 (27.3)	352 (53.3)	309 (46.7)			309 (46.7)	352 (53.3)			355 (53.7)	306 (46.3)		
2001–3000 RMB	672 (27.8)	354 (52.7)	318 (47.3)			318 (47.3)	354 (52.7)			373 (55.5)	299 (44.5)		
3001–4000 RMB	530 (21.9)	259 (48.9)	271 (51.1)			262 (49.4)	268 (50.6)			272 (51.3)	258 (48.7)		
4001–5000 RMB	241 (10.0)	139 (57.7)	102 (42.3)			132 (54.8)	109 (45.2)			136 (56.4)	105 (43.6)		
>5000 RMB	131 (5.4)	75 (57.3)	56 (42.7)			76 (58.0)	55 (42.0)			78 (59.5)	53 (40.5)		
*Marital status*													
Unmarried	280 (11.6)	151 (53.9)	129 (46.1)	*χ* ^2^: 2.043	0.360	161 (57.5)	119 (42.5)	*χ* ^2^: 5.767	0.056	111 (39.6)	169 (60.4)	*χ* ^2^: 4.755	0.093
Married	2063 (85.4)	1072 (52.0)	991 (48.0)			1040 (50.4)	1023 (49.6)			800 (38.8)	1263 (61.2)		
Divorced	74 (3.0)	33 (44.6)	41 (55.4)			34 (45.9)	40 (54.1)			38 (51.4)	36 (48.6)		
*Hospitalizations per year*													
0	1466 (60.7)	743 (50.7)	723 (49.3)	*χ* ^2^: 6.294	0.178	700 (47.7)	766 (52.3)	*χ* ^2^: 4.159	0.385	839 (57.2)	627 (42.8)	*χ* ^2^: 817	0.146
1	599 (24.8)	271 (45.2)	328 (54.8)			265 (44.2)	334 (55.8)			344 (57.4)	255 (42.6)		
2–3	300 (12.4)	138 (46.0)	162 (54.0)			133 (44.3)	167 (55.7)			190 (63.3)	110 (36.7)		
4–6	38 (1.6)	17 (44.7)	21 (55.3)			14 (36.8)	24 (63.2)			20 (52.6)	18 (47.4)		
>6	14 (0.6)	7 (50.0)	7 (50.0)			6 (42.9)	8 (57.1)			11 (78.6)	3 (21.4)		
*Medical insurance*													
Medical insurance in urban area	746 (30.9)	404 (54.2)	342 (45.8)	*χ* ^2^: 9.963	0.076	366 (49.1)	380 (50.9)	*χ* ^2^: 9.805	0.081	332 (44.5)	414 (55.5)	*χ* ^2^: 6.795	0.236
Urban employees' medical insurance	1025 (42.4)	563 (54.9)	462 (45.1)			485 (47.3)	540 (52.7)			498 (48.6)	527 (51.4)		
New-style rural cooperative medical care	404 (16.7)	224 (55.4)	180 (44.6)			204 (50.5)	200 (49.5)			197 (48.8)	207 (51.2)		
Commercial medical insurance	38 (1.6)	17 (44.7)	21 (55.3)			22 (57.9)	16 (42.1)			23 (60.5)	15 (39.5)		
Private expenses	167 (6.9)	110 (65.9)	57 (34.1)			86 (51.5)	81 (48.5)			85 (50.9)	82 (49.1)		
Others	37 (1.5)	22 (59.5)	15 (40.5)			26 (70.3)	11 (29.7)			17 (45.9)	20 (54.1)		
*Adverse reactions*													
Yes	527 (21.8)	276 (52.4)	251 (47.6)	*χ* ^2^: 4.247	0.120	259 (49.1)	268 (50.9)	*χ* ^2^: 4.118	0.128	294 (55.8)	233 (44.2)	*χ* ^2^: 1.160	0.560
No	1417 (58.6)	769 (54.3)	648 (45.7)			688 (48.6)	729 (51.4)			763 (53.8)	654 (46.2)		
Uncertain	473 (19.6)	231 (48.8)	242 (51.2)			206 (43.6)	267 (56.4)			248 (52.4)	225 (47.6)		
*Smoking status*													
No smoking	1781 (73.7)	887 (49.8)	894 (50.2)	*χ* ^2^: 6.034	0.303	847 (47.6)	934 (52.4)	*χ* ^2^: 69.326	<0.001	901 (50.6)	880 (49.4)	*χ* ^2^: 5.740	0.332
Light smoking (PI ≤ 200)	346 (14.3)	154 (44.5)	192 (55.5)			210 (60.7)	136 (39.3)			169 (48.8)	177 (51.2)		
Medium-heavy smoking (PI 200–400)	199 (8.2)	96 (48.2)	103 (51.8)			138 (69.3)	61 (30.7)			91 (45.7)	108 (54.3)		
Heavy smoking (PI ≥ 400)	30 (1.2)	12 (40.0)	18 (60.0)			22 (73.3)	8 (26.7)			18 (60.0)	12 (40.0)		
Smoking cessation	38 (1.6)	19 (50.0)	19 (50.0)			29 (76.3)	9 (23.7)			14 (36.8)	24 (63.2)		
Smoking cessation (≥2 years)	23 (1.0)	8 (34.8)	15 (65.2)			18 (78.3)	5 (21.7)			12 (52.2)	11 (47.8)		
*Alcohol consumption status*													
No drinking	1609 (66.6)	872 (54.2)	737 (45.8)	^a^5.353	0.370	791 (49.2)	818 (50.8)	^a^Fisher: 27.181	<0.001	898 (55.8)	711 (44.2)	^a^Fisher: 6.321	0.264
Heavy drinking	52 (2.2)	24 (46.2)	28 (53.8)			36 (69.2)	16 (30.8)			23 (44.2)	29 (55.8)		
Medium-heavy drinking	232 (9.6)	132 (56.9)	100 (43.1)			148 (63.8)	84 (36.2)			120 (51.7)	112 (48.3)		
Light drinking	512 (21.2)	282 (55.1)	230 (44.9)			281 (54.9)	231 (45.1)			263 (51.4)	249 (48.6)		
Temperance	9 (0.4)	5 (55.6)	4 (44.4)			6 (66.7)	3 (33.3)			5 (55.6)	4 (44.4)		
Temperance (≥2 years)	3 (0.1)	0 (0.0)	3 (100.0)			2 (66.7)	1 (33.3)			2 (66.7)	1 (33.3)		
*Types of medicines per day*													
≤2	1811 (74.9)	981 (54.2)	830 (45.8)	*χ* ^2^: 6.414	0.093	890 (49.1)	921 (50.9)	*χ* ^2^: 6.178	0.103	864 (47.7)	947 (52.3)	^a^Fisher: 9.266	0.026
2–5	570 (23.6)	278 (48.8)	292 (51.2)			248 (43.5)	322 (56.5)			232 (40.7)	338 (59.3)		
6–10	32 (1.3)	16 (50.0)	16 (50.0)			13 (40.6)	19 (59.4)			15 (46.9)	17 (53.1)		
>10	4 (0.2)	1 (25.0)	3 (75.0)			2 (50.0)	2 (50.0)			1 (25.0)	3 (75.0)		
*Number of medications received each time*													
≤5	1986 (82.2)	1066 (53.7)	920 (46.3)	*χ* ^2^: 5.458	0.142	971 (48.9)	1015 (51.1)	*χ* ^2^: 1.343	0.719	938 (47.2)	1048 (52.8)	*χ* ^2^: 13.317	0.004
6–10	372 (15.4)	176 (47.3)	196 (52.7)			172 (46.2)	200 (53.8)			153 (41.1)	219 (58.9)		
11–15	45 (1.9)	22 (48.9)	23 (51.1)			20 (44.4)	25 (55.6)			20 (44.4)	25 (55.6)		
>15	14 (0.6)	8 (57.1)	6 (42.9)			6 (42.9)	8 (57.1)			1 (7.1)	13 (92.9)		
*Self-rated health status*													
Very good	336	181 (53.9)	155 (46.1)	*χ* ^2^: 7.936	0.094	164 (48.8)	172 (51.2)	*χ* ^2^: 5.637	0.228	195 (58.0)	141 (42.0)	*χ* ^2^: 37.000	<0.001
Good	944	519 (55.0)	425 (45.0)			495 (52.4)	449 (47.6)			449 (47.6)	495 (52.4)		
Fair	924	475 (51.4)	449 (48.6)			496 (53.7)	428 (46.3)			394 (42.6)	530 (57.4)		
Poor	198	97 (49.0)	101 (51.0)			98 (49.5)	100 (50.5)			71 (35.9)	127 (64.1)		
Very poor	15	4 (26.7)	11 (73.3)			11 (73.3)	4 (26.7)			3 (20.0)	12 (80.0)		

Abbreviations: CRA: community residents' administration of medicine; CRR: community residents' recognition of medicines; CRC: community residents' medication compliance; *χ*^2^: chi-squared; ^a^Fisher's exact text was conducted.

**Table 3:** Mann-Whitney *U* test analyses of continuous independent variables versus poor and better CRA, CRR, and CRC.

Variables	CRA				CRR				CRC			
	Appropriately *N* (%)	Inappropriately *N* (%)	Statistics	*p*	Appropriately *N* (%)	Inappropriately *N* (%)	Statistics	*p*	Appropriately *N* (%)	Inappropriately *N* (%)	Statistics	*p*
Age (years)	1141 (47.2)	1276 (52.8)	*Z* = −5.387	<0.001	1264 (52.3)	1153 (47.7)	*Z* = −6.929	<0.001	1112 (46.0)	1305 (54.0)	*Z* = −6.195	<0.001

Abbreviations: CRA: community residents' administration of medicine; CRR: community residents' recognition of medicines; CRC: community residents' medication compliance; *χ*^2^: chi-squared.

**Table 4:** Multivariate logistic regression on association between selected factors and poor and better CRA, CRR, and CRC.

Variables	Logistic regression analysis								
	CRA			CRR			CRC		
	Odds ratio	95% CI	*p*	Odds ratio	95% CI	*p*	Odds ratio	95% CI	*p*
*Gender*									
Male	1			1			1		
Female	1.469	1.231–1.753	<0.001	1.100	0.897–1.348	0.361	1.258	1.059–1.495	0.009
*Age (years)*									
	0.997	0.990–1.004	0.394	0.987	0.981–0.994	<0.001	1.013	1.005–1.020	0.001
*Highest educational level*									
Primary school	1			1			^a^NA		
Middle school	0.997	0.694–1.432	0.985	0.763	0.528–1.102	0.149			
Senior high school or polytechnic	1.437	0.979–2.110	0.064	1.067	0.728–1.564	0.740			
Junior college	1.598	1.045–2.444	0.031	1.527	1.006–2.317	0.047			
Undergraduate	2.627	1.619–4.263	<0.001	1.505	0.948–2.389	0.043			
*Occupation*									
Agricultural worker	1			1			1		
Industrial (or skilled) worker	1.249	0.862–1.809	0.240	0.983	0.702–1.376	0.921	1.432	1.003–2.044	0.048
Professional	1.688	1.089–2.617	0.019	1.267	0.847–1.897	0.250	1.814	1.189–2.769	0.006
Service provider	1.506	1.017–2.229	0.041	1.037	0.717–1.501	0.846	1.231	0.843–1.798	0.283
Other	1.398	0.947–2.065	0.092	1.075	0.740–1.561	0.705	1.312	0.895–1.922	0.164
*Medical insurance*									
Medical insurance in urban area	^a^NA			^a^NA			1		
Urban employees' medical insurance							1.121	0.918–1.369	0.264
New-style rural cooperative medical care							0.865	0.654–1.144	0.310
Commercial medical insurance							0.591	0.299–1.166	0.129
Private expenses							1.046	0.735–1.489	0.803
Others							1.163	0.590–2.292	0.604
*Smoking status*									
No smoking	^a^NA			1			^a^NA		
Light smoking (PI ≤ 200)				0.684	0.518–0.904	0.008			
Middle-heavy smoking (PI 200–400)				0.463	0.317–0.676	<0.001			
Heavy smoking (PI ≥ 400)				0.378	0.162–0.885	0.025			
Smoking cessation				0.313	0.141–0.694	0.004			
Smoking cessation (≥2 years)				0.330	0.115–0.945	0.039			
*Alcohol consumption status*									
No drinking	^a^NA			1			^a^NA		
Heavy drinking				0.664	0.352–1.252	0.206			
Medium-heavy drinking				0.854	0.598–1.219	0.384			
Light drinking				0.926	0.735–1.167	0.515			
Temperance				1.221	0.270–5.518	0.796			
Temperance (≥2 years)				0.775	0.054–11.091	0.851			
*Types of medicines per day*									
≤2	^a^NA			^a^NA			1		
2–5							0.961	0.742–1.244	0.761
6–10							0.702	0.320–1.538	0.376
>10							1.934	0.171–21.847	0.594
*Number of medications received each time*									
≤5	^a^NA			^a^NA			1		
6–10							1.035	0.776–1.382	0.813
11–15							0.814	0.422–1.570	0.538
15							7.436	0.940–58.844	0.057
*Self-rated health status*									
Very good	^a^NA			1			1		
Good				1.081	0.831–1.405	0.563	1.458	1.126–1.888	0.004
Fair				1.219	0.928–1.602	0.154	1.693	1.293–2.217	<0.001
Poor				1.821	1.239–2.678	0.002	2.064	1.377–3.094	<0.001
Very poor				0.779	0.225–2.700	0.694	5.388	1.408–20.612	0.014

Abbreviations: CRA: community residents' administration of medicine; CRR: community residents' recognition of medicines; CRC: community residents' medication compliance; *χ*^2^: chi-squared; NA: not applicable.^a^No relationship in independent variables from chi-squared (*χ*^2^) analyses or Fisher's exact test and Mann-Whitney *U* test.

## Data Availability

The datasets generated and analyzed during the present study are available from the corresponding author on reasonable request.

## Abbreviations

CRA: Community residents' administration of medicine
CRR: Community residents' recognition of medicines
CRC: Community residents' medication compliance
*χ*^2^: Chi-squared.
